# Adaptations of evidence-based trauma-focused interventions for children and adolescents: a systematic review

**DOI:** 10.1186/s43058-022-00348-5

**Published:** 2022-10-08

**Authors:** Brittany C. L. Lange, Ashley Nelson, Jason M. Lang, Shannon Wiltsey Stirman

**Affiliations:** 1 Child Health and Development Institute of Connecticut, Inc., Farmington, USA; 2 Department of Psychiatry, UCONN Health, Farmington, USA; 3 Child Study Center, Yale School of Medicine, New Haven, USA; 4 National Center for PTSD, Washington, D.C., USA; 5 Stanford University, Stanford, USA

**Keywords:** Systematic review, Evidence-based interventions, Interventions, Adaptation, Traumatic events, Trauma, Mental health, Children, Adolescents

## Abstract

**Background:**

Rates of potentially traumatic events (PTEs) and other forms of adversity among children are high globally, resulting in the development of a number of evidence-based interventions (EBIs) to address the adverse outcomes stemming from these experiences. Though EBIs are intended to be delivered according to set parameters, these EBIs are frequently adapted. However, little is known about existing adaptations of EBIs for children who experienced PTEs or other adversities. As such, this review aimed to determine: (1) why existing EBIs designed to address PTEs and other adversities experienced by children are adapted, (2) what processes are used to determine what elements should be adapted, and (3) what components of the intervention are adapted.

**Methods:**

Nine academic databases and publicly available search engines were used to identify academic and grey literature. Initial screening, full-text review, data extraction, and quality determinations were completed by two members of the research team. Data were synthesized narratively for each adapted EBI by research question.

**Results:**

Forty-two studies examining the adaptations of nine different EBIs were located, with Trauma-Focused Cognitive Behavioral Therapy and Cognitive Behavioral Intervention for Trauma in Schools being the most commonly adapted EBIs. Most frequently, EBIs were adapted to improve fit with a new population and to address cultural factors. Most commonly, researchers in combination with others made decisions about adapting interventions, though frequently who was involved in these decisions was not described. Common content adaptations included the addition of intervention elements and the tailoring/tweaking/refining of intervention materials. Common contextual adaptations included changes to the intended population, changes to the channel of treatment delivery, and changes to who administered the intervention.

**Conclusions:**

Most published studies of EBI adaptions have been developed to improve fit and address cultural factors, but little research is available about adaptations made by clinicians in day-to-day practice. Efforts should be made to evaluate the various types of adaptations and especially whether adaptations improve access to services or improve child outcomes in order to ensure that all children exposed to trauma can access effective treatment.

**Trial registration:**

The protocol for this systematic review was published with PROSPERO (CRD42020149536).

**Supplementary Information:**

The online version contains supplementary material available at 10.1186/s43058-022-00348-5.

Contributions to the literature

- Researchers often adapt evidence-based interventions (EBIs) for children who have experienced trauma. However, little is known about the process of adapting these interventions or what adaptations are made.
- This review’s findings contribute to the existing literature by synthesizing information on how EBIs for children who experienced trauma are adapted, who makes the decision to adapt these interventions, and what components are adapted.
- Recommendations for enhancing research on EBI adaptations are provided, including evaluating adaptations to determine whether these adaptations improve access to services or improve child outcomes.

## Background

Childhood exposure to potentially traumatic events (PTEs) and other forms of adversity represents a significant global public health problem. Examples of PTEs include experiencing violence, physical abuse, sexual abuse, a serious accident, serious physical illness, the death of someone close, or a natural disaster [1]. Adversity is conceptualized more broadly than PTEs and includes other forms of stressful or difficult circumstances such as poverty, discrimination, living with a caregiver who has mental illness or substance use problems, and other forms of adverse childhood experiences (ACEs [2, 3]). Recent research has demonstrated that PTEs and other forms of adversity are highly prevalent among children. Specifically, in a study of 4000 children conducted in the USA, 60.8% of children had experienced direct exposure to violence, abuse, or crime within the past year [4].

If PTEs or other forms of adversity are not addressed, these events can have negative effects into adulthood, including poorer mental and physical health, and increased risky substance use and sexual behaviors [5]. Given high rates of PTEs and subsequent negative outcomes stemming from these events, a number of evidence-based interventions (EBIs) have been developed for children, such as Trauma-Focused Cognitive Behavioral Therapy (TF-CBT), Cognitive Behavioral Therapy, and Child Parent Psychotherapy [6].

Existing EBIs have been modified or adapted for a number of reasons. A recent global systematic review of public health interventions found that the most common reasons for adapting interventions were “the need for cultural appropriateness (64.3%), focusing on a new target population (59.5%), and implementing in a new setting (57.1%). Common adaptations were content (100%), context (95.2%), cultural modifications (73.8%), and delivery (61.9%)” [7].

Given the breadth of available EBIs, systematic reviews have been conducted to examine these interventions. One systematic review examining EBI adaptations did not focus on EBIs to treat child traumatic stress [7], while others have examined EBIs and other interventions for children who have experienced traumatic stress, without discussing the nature of included adapted interventions in depth or while focusing on a specific target population [8–11]. These reviews note that understanding the types of adaptations that are made is an important area for further research. Thus, this review aims to answer the following research questions:

1. Why are existing EBIs designed to address PTEs and other forms of adversity experienced by children being adapted?
2. What processes are used to determine which elements of the intervention should be adapted?
3. What components of the intervention are adapted?

## Methods

### Pre-registration and reporting guidelines

The protocol for this review and subsequent amendments to this review (e.g., minor updates to research questions) were published with PROSPERO [12]. This systematic review adheres to the PRISMA 2020 guidelines for reporting systematic reviews [13] and the PRISMA 2020 Checklist for systematic reviews is included as an Additional file 1.

### Search strategy

The full search strategy for this review is available in Additional file 2. Nine academic databases were searched on October 14, 2019: Applied Social Sciences Index & Abstracts, Embase, ProQuest Dissertations & Theses Global, PsycINFO, PubMed, Scopus, Social Services Abstracts, Sociological Abstracts, and Web of Science. The search strategy was created by compiling text words and controlled vocabularies (when allowed by the database) within each database that covered four areas: evidence-based interventions, trauma, the population of interest (i.e., children and adolescents), and adaptation. The search was validated using a list of pre-identified studies meeting the inclusion criteria for this review. Pre-identified studies were located by searching the names of several existing EBIs developed to address trauma, which were located through the National Child Traumatic Stress Network’s list of interventions for trauma [14], and the term “adaptation.” Additionally, a supplementary search was conducted on August 31, 2021, in PubMed using the same search terms as the original search to capture studies that may have been published since the original search date. Both searches were developed and run by the first author (BL).

In addition to academic databases, Google was used to identify potentially relevant grey literature. The first author (BL) searched Google using different combinations of terms used in the academic databases and reviewed the first three-five pages of results for potentially relevant articles for each set of search terms. Further, a list of conference presentations on EBI adaptations was compiled from the National Child Traumatic Stress Network’s list of interventions for trauma [14]. The authors of these presentations were contacted to determine if manuscripts or reports had been written based on the presentations.

Once included studies were determined from the initial full-text review (process outlined below), all included studies had their references searched to identify additional potential studies for inclusion. Further, all included studies were forward searched (i.e., studies that cited included studies were screened for potential inclusion). This process was repeated for any study found through this method until no new studies could be located. In both instances, the first author (BL) completed the search, with all potential new articles for inclusion being reviewed by the second author (AN).

### Screening based on study inclusion and exclusion criteria

Studies located through searches in academic databases and non-academic search engines were downloaded to EndNote where duplicates were removed. Following this, the remaining studies were uploaded to Rayyan (an online tool for systematic review management) for screening [15]. During the initial screening, which occurred at the title and abstract level, the first author (BL) screened 100% of the studies, while the second author (AN) screened 20% of the studies. This percentage was purposively chosen based on the methods of other high-quality systematic reviews. The goal of this initial screening was to briefly assess studies to determine if there was any likelihood they may be relevant to the review, with both authors prioritizing sensitivity in screening to ensure that no potentially relevant studies were missed. Upon completion of this screening, both authors met to discuss and resolve discrepancies. Once all discrepancies were resolved, full texts of the remaining studies were located. These full texts were screened using the same process as the initial screening (i.e., BL reviewed 100% of full texts, and AN reviewed 20% of full texts, with discrepancies resolved through further review of full texts and discussion until a consensus could be reached). For each step of the process, the third author (JL) was available to help resolve discrepancies. However, BL and AN were able to resolve all discrepancies without consultation with JL.

To be included in the review, the article had to meet the following criteria:

1. A full text is available.
2. Is in English. Articles not published in English were excluded, as there were no resources available for translation of articles.
3. Is empirical. Articles with an abstract only, reviews, or think pieces were excluded.
4. Evaluates an intervention that was developed to address PTEs and traumatic stress or has been adapted to address PTEs and traumatic stress.
5. Evaluates an adaptation of an evidence-based intervention. There are different conceptualizations of what constitutes an “evidence-based” intervention. For this review, an intervention was considered “evidence-based” if it was registered with the California Evidence-Based Clearinghouse [16] or Blueprints for Healthy Youth Development [17].
6. Evaluates an EBI for children (18 and under). This EBI could have been developed specifically for children or adapted for use with children.
7. The adaptation to the EBI is clearly described and provided similarly to all children in the study.
8. Contains at least one child mental health outcome.
9. Does not contain solely case studies.

In instances where both a thesis and journal article are available about the same study, only the journal article was included. Further, in instances where primary data is duplicated in multiple publications, only one study was included.

### Data extraction

We conducted data extraction in Excel. Items for extraction included the following main topics: basic study information (research question, theoretical framework, etc.), methods (sampling strategy, data analysis, etc.), adaptation reasons (goal of adaptation, type of adaptation), adaptation processes (how decisions to adapt were made, specific adaptations made, etc.), results (participant characteristics; changes in mental health outcomes, such as post-traumatic stress), and next steps (future research and policy implications). Items chosen for extraction related to adaptations were from the Framework for Reporting Adaptations and Modifications – Expanded (FRAME [18]). BL completed extraction for 100% of included studies, while AN completed extraction for 20% of included studies. 20% was selected purposively based on best practices for systematic reviews. All discrepancies in extraction were resolved through further review of the article and discussion among BL and AN. Though JL was available to help address discrepancies if needed, BL and AN were able to address all discrepancies among themselves.

### Quality determinations

To our knowledge, no validated measure exists to assess the risk of bias specifically in studies that have made adaptations. As such, a general measure to assess bias, the Evidence Project Risk of Bias Tool was used [19]. BL and AN conducted risk of bias assessments. Both authors did not have formal training on the use of this measure, but engaged in a thorough review of the literature on the measure to ensure that it was being used appropriately. BL conducted 100% of risk of bias assessments, while AN conducted 20% of risk of bias assessments to ensure reliability. 20% was selected purposively based on best practices for systematic reviews.

### Data Synthesis

For each EBI, data were narratively synthesized for each adapted EBI by research question using the agreed upon extraction documents described previously.

## Results

### Overview of results

**Fig. 1:**
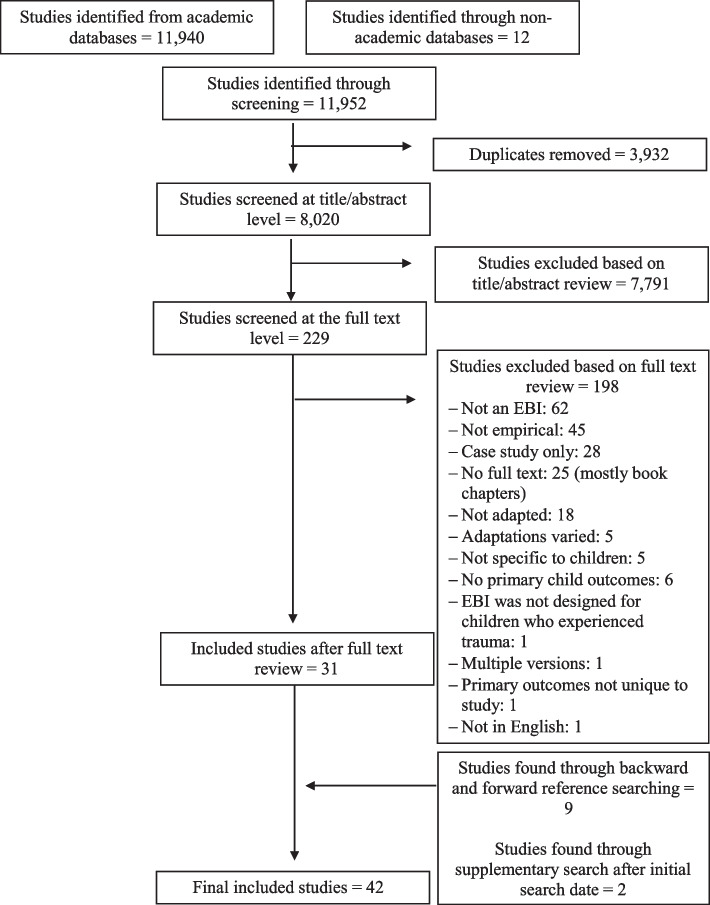
Selection of studies for the review

Forty-two studies examining the adaptations of nine different EBIs were included in this systematic review [20–61]. Please see Fig. 1 for a flow diagram of our review and selection process. Many of these were published between 2011-2015 [21, 22, 24–29, 31, 41–44, 46, 48, 50, 57, 60] and were conducted in the USA [20, 22, 23, 30–42, 45, 46, 48, 49, 57–61]. The average sample size in these studies was 55.3 (SD=51.4). The most commonly adapted EBIs were TF-CBT [20–33, 61] and Cognitive Behavioral Intervention for Trauma in Schools [34–42]. See Table 1.

**Table 1:** Overview of included studies (*N* = 42)^a^

	***n***	%	Mean (SD)
**Full sample size**			55.3 (51.4)
**Date of publication**			
2003–2005	3	7.1	
2006–2010	12	28.6	
2011–2015	18	42.9	
2016–2020	8	19.0	
2021	1	2.4	
**Country**			
USA	25	59.5	
Israel	3	7.1	
Democratic Republic of Congo	2	4.8	
Sri Lanka	2	4.8	
Zambia	2	4.8	
Canada	1	2.4	
Finland	1	2.4	
Germany	1	2.4	
Jordan	1	2.4	
Pakistan	1	2.4	
Somalia	1	2.4	
Tanzania	1	2.4	
Not listed	1	2.4	
**EBI adapted**			
Trauma-Focused Cognitive Behavioral Therapy	15	35.7	
Cognitive Behavioral Intervention for Trauma in Schools	9	21.4	
Prolonged Exposure Therapy	8	19.0	
Narrative Exposure Therapy	5	11.9	
Multisystemic Therapy	1	2.4	
Multisystemic Therapy for Child Abuse and Neglect and Multisystemic Therapy	1	2.4	
Seeking Safety	1	2.4	
Skills Training in Affective and Interpersonal Regulation	1	2.4	
Support for Students Exposed to Trauma	1	2.4	

^a^Percentages may not sum to 100% due to rounding

Across all EBIs, the decision to adapt an intervention was most often made by the researcher in collaboration with others [21, 23, 26–30, 34, 35, 37, 38, 40–49, 56, 57]. The most common reason for adaptations was to improve fit with recipients [20, 23, 24, 34, 43–54, 57–62]. The second most common reason for adaption was to address cultural factors, a subcategory of improving fit [18], to meet the needs of diverse racial and ethnic populations. This was listed as the main reason for adaptation [21, 25, 30, 35–38, 40] or one of the main reasons for adaptation [26–29, 41, 42, 56] in fifteen articles. The most common types of contextual adaptations were changes to the intended population [34, 35, 43–54, 57–60, 62], such as a treatment designed for adults being adapted to children; changes to format [21, 23, 25, 28, 29, 31–33, 37, 39], such as changing the channel of delivery for a particular intervention component from in-person to virtual; and changes in personnel [26–29, 31, 32, 34, 39, 56], such as changing who an intervention is designed to be administered by from a therapist to a lay worker. The most common content adaptations were adding elements [20, 21, 23, 24, 26–29, 31–37, 40–56, 58, 59, 61]; tailoring, tweaking, or refining of materials [21, 23, 25–27, 29, 30, 33–38, 40–52, 56, 58]; shortening or condensing intervention elements [20–22, 41–50, 57]; lengthening or extending intervention elements [21, 26, 34, 35, 37, 38, 41, 42, 58, 59]; and removing or skipping elements [22, 24, 34, 36, 39, 40, 57]. See Table 2.

**Table 2:** Overview of adaptations based on FRAME (*N* = 42)^a^

	***n***	%
**Reason for adaptation**		
Improve fit with recipients	22	52.4
Address cultural factors	8	19.0
Address cultural factors/improve feasibility	3	7.1
Address cultural factors/increase engagement/improve effectiveness	2	4.8
Improve feasibility	2	4.8
Increase reach/improve feasibility/reduce cost	2	4.8
Address cultural factors/increase reach	1	2.4
Address cultural factors/improve feasibility/improve fit	1	2.4
Increase reach and improve feasibility	1	2.4
**Who was involved in adaptation**		
Others and researcher^b^	23	54.8
Not listed	16	38.1
Researcher	3	7.1
**Content adaptations**^**c**^		
Adding elements	35	83.3
Tailoring/tweaking/refining	28	66.7
Shortening/condensing	14	33.3
Lengthening/extending	10	23.8
Removing/skipping elements	7	16.7
Integrating another treatment into EBP	4	9.5
Re-ordering of intervention modules or segments	3	7.1
Spreading	3	7.1
Repeating elements or modules	1	2.4
Integrating the intervention into another approach	1	2.4
**Contextual adaptations**^**c**^		
Population	19	45.2
Format	10	23.8
Personnel	9	21.4
Setting	3	7.1

^a^ Percentages may not sum to 100% due to rounding

^b^ In seven instances, this information was not listed in the article itself, but was found in the only intervention manual available to the research team [63]

^c^ Interventions can have multiple content and contextual adaptations

Results of research questions are summarized by EBI below, with detailed information on each EBI presented in Tables 3, 4, 5, 6, and 7. Though FRAME includes adaptations made to Training and Evaluation, and Implementation and Scale-Up, these were infrequently made or not reported on by study authors. Thus, Tables 3, 4, 5, 6, and 7, and the summaries below focus on content and contextual adaptations. See Additional file 3 for study quality ratings.

**Table 3:** Adaptations of TF-CBT

Study author, year	Country	Population	Reason for adaptation	Who was involved in adaptation	FRAME categories	Examples of content adaptations	Examples of contextual adaptations
Cohen et al., 2004 [61]	USA	6–17-year-old children who lost a loved one	Improve fit	Not listed	− Adding elements	− Added grief-focused components	None
Cohen et al., 2006 [20]	USA	6–17-year-old children who experienced traumatic loss	Improve fit	Not listed	− Adding elements − Shortening/condensing	− Used modified protocol (specific to traumatic loss) that included grief-focused components − Decreased sessions of CBT-CTG by compressing grief module	None
Damra et al., 2014 [21]	Jordan	10–12-year-old boys who experienced physical abuse	Address cultural factors	Researchers and expert clinicians	− Adding elements − Format − Lengthening/extending − Shortening/condensing − Tailoring/tweaking/refining	− Translated into Arabic − Parents attended Better Parenting Skills Education − Decreased number of sessions − Increased length of sessions	− Changed format to group delivery
Deblinger et al., 2011 [22]	USA	4–11-year-old children who experienced contact sexual abuse	Improve feasibility	Not listed	− Removing/skipping elements − Shortening/condensing	− Decreased sessions − Removed trauma narrative	None
Heier, 2019 [23]	USA	15–18-year-old children in the juvenile justice system	Improve fit	Administrators, researchers, and individual practitioners	− Adding elements − Format - channel of delivery − Re-ordering of intervention modules or segments − Tailoring/tweaking/refining	− Ability to re-order elements − Added strategies to maintain therapeutic alliance − Added education of correctional staff − Modified strategies for imaginal desensitization	− Parents could be included in session through technology (phone or videoconferencing)
Madigan et al., 2015 [24]	Not listed	12–18-year-old pregnant girls who experienced traumatic loss	Improve fit	Researchers – theoretically driven	− Adding elements − Removing/skipping elements	− Used modified protocol (specific to traumatic loss) − Removed parent sessions	None
McMullen et al., 2013 [25]	DRC	13–17-year-old boys who were affected by war	Address cultural factors	Not listed	− Format − Tailoring/tweaking/refining	− Included culturally applicable examples, analogies, songs, and stories	− Changed format to group delivery
Murray et al., 2015^a^ [27]	Zambia	5–18-year-old children who experienced trauma	Address cultural factors and improve feasibility	Researchers, local counselors, and community members	− Adding elements − Personnel − Tailoring/tweaking/refining	− Included culturally applicable analogies, stories, values (e.g., religious), and items − Simplified language − Included multiple caregivers from the family system	− Administered by lay counselors
Murray et al., 2013 [26]	Zambia	5–18-year-old children who experienced trauma	Address cultural factors and improve feasibility	Researchers, local counselors, and community members	− Adding elements − Lengthening/extending − Personnel − Tailoring/tweaking/refining	− Included culturally applicable analogies, stories, values (e.g., religious), and items − Simplified language − Included multiple caregivers from the family system − Lengthened sessions to up to 2 hrs based on client preference	− Administered by lay counselors
O’Callaghan et al., 2013 [29]	DRC	12–17-year-old girls who were affected by war and experienced or witnessed rape or sexual abuse	Address cultural factors and improve feasibility	Individual practitioners and researchers	− Adding elements − Format − Personnel − Tailoring/tweaking/refining	− Included culturally appropriate examples, games, and songs − Included culturally appropriate ways to reduce risk of sexual violence − Social worker visited family to facilitate relationship between child and family	− Administered by non-clinicians − Changed format to group delivery for most modules
O'Donnell et al., 2014 [28]	Tanzania	6–13-year-old children who lost a parent	Address cultural factors, improve feasibility, and improve fit	Researchers and community members	− Adding elements − Format − Personnel	− Used modified protocol (specific to traumatic loss) − Individual child/caregiver sessions added for creation of narrative	− Administered by lay counselors − Changed format to group delivery
Rivera, 2008 [30]	USA	7–17-year-old Hispanic children who experienced trauma	Address cultural factors	Researchers, clinicians, intervention developers, mental health experts, and community members	− Tailoring/tweaking/refining	− Included cultural constructs (e.g., machismo, familismo) − Modified treatment components to make them more acceptable (e.g., integrated spirituality) − Modified the trauma narrative to include challenges related to lack of closure − Included culturally appropriate examples	None
Salloum et al., 2014 [31]	USA	3–6-year-old children who experienced trauma	Increase reach, improve feasibility, and reduce cost	Not listed	− Adding elements − Format - channel of delivery − Integrating another approach into treatment − Personnel	− Added caregiver-child workbook, which was based on another treatment approach	− Delivered in a two-step model − Step 1 is caregiver-led and not clinician-led − Delivered some material through phone meetings/a website
Salloum et al., 2017 [32]	USA	8–12-year-old children who experienced trauma	Increase reach, improve feasibility, and reduce cost	Not listed	− Adding elements − Format - channel of delivery − Integrating another approach into treatment − Personnel	− Added caregiver-child workbook, which was based on another treatment approach	− Delivered in a two-step model − Step 1 is caregiver-led and not clinician-led − Delivered some material through phone meetings/a website
Stewart et al., 2017 [33]	USA	7–16-year-old children who experienced trauma	Increase reach and improve feasibility	Not listed	− Adding elements − Format - channel of delivery − Tailoring/tweaking/refining	− Materials presented through technology (e.g., PowerPoint, digital books, and writing of narrative in Word) − Addition of digital materials (e.g., games and books)	− Changed format to telehealth

^a^ Information on adaptations obtained from Murray et al. [26] who was cited by Murray et al. [27]

**Table 4:** Adaptations of Cognitive Behavioral Intervention for Trauma in Schools

Study author, year	Country	Population	Reason for adaptation	Who was involved in adaptation	FRAME categories	Content adaptations	Contextual adaptations
Auslander et al., 2017 [34]	USA	12–18-year-old girls in child welfare who experienced trauma	Improve fit	Intervention developer/purveyor, practitioners, researchers, and recipients	− Adding elements − Lengthening/extending − Personnel − Population − Removing/skipping elements − Setting − Tailoring/tweaking/refining	− Lengthened sessions − Added sessions (pre-intervention and graduation) − Removed teacher session − Included population appropriate language and examples − Added grounding/relaxation to each session − Added reminders for meetings by phone	− Changed setting to child welfare − Population changed (12-18 year olds, girls, in child welfare, and could have experienced sexual abuse) − Two group facilitators
Elswick et al., 2021 [35]	USA	12–18-year-old African refugee children who experienced trauma	Address cultural factors	Researchers with parent and participant feedback	− Adding elements − Lengthening/extending − Population − Setting − Tailoring/tweaking/refining	− Lengthened intervention − Parent support groups offered included cultural brokers and interpreters − Modified language (e.g., did not use the term “homework”) − Added culturally appropriate activities (e.g., drumming) − Incorporated a pyramid mentoring model	− Changed setting to community − Population changed (12-18 year olds) − Delivered by participant gender (no option of mixed-gender groups)
Feldman, 2007 [36]	USA	Spanish speaking, immigrant children in middle school who experienced trauma	Address cultural factors	Researchers	− Adding elements − Removing/skipping elements − Tailoring/tweaking/refining	− Ran sessions bilingually − Included culturally appropriate examples − Removed some program components − Addition of communication with families (e.g., could meet with group leaders, phone calls made)	None
Goodkind et al., 2010 [37]	USA	12–15-year-old American Indian children in school who experienced trauma	Address cultural factors	Researchers, community members, and clinicians	− Adding elements − Format − Lengthening/extending − Re-ordering of intervention modules or segments − Spreading − Tailoring/tweaking/refining	− Increased sessions (split one session into two) − Changed timing of parent sessions − Included culturally appropriate examples and stories and removed inappropriate ones − Added elements (e.g., alternative activities, option to see traditional healer) − Timing of parent session changed	− Individual (non-group) time spent with students to identify supportive person
Jaycox et al., 2009 [39]	USA	11.5-year-old (on average) children in school who experienced severe violence	Improve feasibility	Not listed	− Format − Personnel − Removing/skipping elements	− Removed sessions (break-out and parent sessions)	− Administered by school personnel − Changed session format to lesson plan format − Changed imaginal exposure to curricular format
Kataoka et al., 2003 [38]	USA	11.4-year-old (on average) Latino, immigrant children in school who experienced community violence	Address cultural factors	Researchers, community members, and clinicians	− Lengthening/extending − Tailoring/tweaking/refining	− Increased family sessions (four 2-h optional multifamily group sessions offered) − Multifamily groups sessions included support for common experiences related to immigration	None
Morsette et al., 2009 [40]	USA	11–12-year-old American Indian children in school who experienced trauma	Address cultural factors	Researchers, community members, and clinicians	− Adding elements − Removing/skipping elements − Tailoring/tweaking/refining	− Included culturally appropriate examples − Removed introductory activities and non-culturally appropriate materials − Modified hot seat activity − Elder provided prayer and participated in graduation	None
Santiago et al., 2014^a^ [41]	USA	11.7-year-old (on average) children in school who experienced trauma	Address cultural factors, increase engagement, and improve effectiveness	Researchers, community members, and clinicians	− Adding elements − Integrating another approach into treatment − Lengthening/extending − Shortening/condensing − Spreading − Tailoring/tweaking/refining	− Family component required − Added material to improve parent functioning − The time spent on each module increased for families − Content put into modules that could be grouped or stretched out − Included culturally appropriate examples and examples appropriate to low-income families − Added adapted material from other approaches (e.g., The Incredible Years)	None
Santiago et al., 2015 [42]	USA	11.8-year-old (on average) children in school who experienced trauma	Address cultural factors, increase engagement, and improve effectiveness	Researchers, community members, and clinicians	− Adding elements − Integrating another approach into treatment − Lengthening/extending − Shortening/condensing − Spreading − Tailoring/tweaking/refining	− Family component required − Added material to improve parent functioning − The time spent on each module increased for families − Content put into modules that could be grouped or stretched out − Included culturally appropriate examples and examples appropriate to low-income families − Added adapted material from other approaches (e.g., The Incredible Years)	None

^a^ Information on some adaptations obtained from Santiago et al. [42] who was cited by Santiago et al. [41]

**Table 5:** Adaptations of Prolonged Exposure Therapy

Study author, year	Country	Population	Reason for adaptation	Who was involved in adaptation	FRAME categories	Content adaptations	Contextual adaptations
Aderka, Appelbaum-Namdar, et al., 2011 [44]	Israel	8–17-year-old children who experienced trauma	Improve fit	Researchers, therapists, and community members	− Adding elements − Population − Shortening/condensing − Tailoring/tweaking/refining	− Added developmentally appropriate activities (e.g., inclusion of drawing, writing, and games) − Involved caregivers − Option included to replace or augment procedures based on the developmental/emotional needs of the adolescent − Simplified language and material for younger adolescents − Session time decreased or breaks given for younger adolescents − Social/developmental challenges faced by adolescents incorporated	− Population changed to children
Aderka, Foa, et al., 2011 [43]	Israel	8–18-year-old children who experienced trauma	Improve fit	Researchers, therapists, and community members			
Brown et al., 2019 [45]	USA	15.3-year-old (on average) girls who experienced sexual assault	Improve fit	Researchers, therapists, and community members			
Foa et al., 2013 [46]	USA	15.3-year-old (on average) girls who experienced sexual abuse	Improve fit	Researchers, therapists, and community members			
Gilboa-Schechtman et al., 2010 [47]	Israel	12–18-year-old children who experienced a single traumatic event	Improve fit	Researchers, therapists, and community members			
McLean et al., 2015 [48]	USA	13–18-year-old girls who experienced sexual assault	Improve fit	Researchers, therapists, and community members			
McLean et al., 2017 [49]	USA	13–18-year-old girls who experienced sexual assault	Improve fit	Researchers, therapists, and community members			
Adler Nevo & Manassis, 2011 [50]	Canada	10.8-year-old (on average) children who experienced trauma	Improve fit	Not listed	− Adding elements − Population − Shortening/condensing − Tailoring/tweaking/refining	− Added activities (e.g., drawing and playing), which could be tailored based on the age of the child − Involved caregivers − Modular rather than session-based, so multiple modules could be presented in one session	− Population changed to children

**Table 6:** Adaptations of Narrative Exposure Therapy

Study author, year	Country	Population	Reason for adaptation	Who was involved in adaptation	FRAME categories	Content adaptations	Contextual adaptations
Catani et al., 2009^a^ [51]	Sri Lanka	8–14-year-old children who experienced war and a tsunami	Improve fit	Not listed	− Adding elements − Population − Tailoring/tweaking/refining	− Used age-appropriate metaphors − Illustrative, creative elements added (e.g., stones, flowers, lifeline, and drawings) − Added body positioning and toys to aid in reenactment	− Population changed to children
Onyut et al., 2005 [52]	Somalia	12–17-year-old children who experienced war	Improve fit	Not listed	− Adding elements − Population − Tailoring/tweaking/refining	− Illustrative, creative methods added (e.g., stones, flowers, lifeline, and drawings) − Narrative was extended beyond the present	− Population changed to children
Peltonen & Kangaslampi, 2019 [53]	Finland	9–17-year-old children who experienced family violence or were refugees	Improve fit	Not listed	− Adding elements − Population	− Illustrative, creative elements added (e.g., lifeline)	− Population changed to children
Ruf et al., 2010 [54]	Germany	7–16-year-old refugee children who experienced trauma	Improve fit	Not listed	− Adding elements − Population	− Illustrative, creative elements added (e.g., stones, flowers, lifeline, and drawings) − Added body positioning to aid in reenactment	− Population changed to children
Schauer, 2008 [55]	Sri Lanka	6–15-year-old children who experienced trauma	Improve fit	Not listed	− Adding elements − Population	− Illustrative, creative elements added (e.g., stones, flowers, lifeline, and drawings) − Added body positioning and toys to aid in reenactment	− Population changed to children

^a^ Information on content/contextual adaptations obtained from Neuner et al. [64] who was cited by Catani et al. [51], as they did not describe the adaptations in the article

**Table 7:** Adaptations of Other EBIs

Study author, year	EBI	Country	Population	Reason for adaptation	Who was involved in adaptation	FRAME categories	Content adaptations	Contextual adaptations
Amin et al., 2020 [56]	SSET	Pakistan	11.4-year-old (on average) children who experienced trauma	Address cultural factors and increase reach	Researcher and community members	− Adding elements − Personnel − Tailoring/tweaking/refining	− Materials translated − Regular parent meetings were scheduled	− Led by individual with clinical training
Gudiño et al., 2014 [57]	STAIR	USA	12–17-year-old children who experienced trauma and were in inpatient treatment	Improve fit	Researchers and clinicians	− Population − Removing/skipping elements − Re-ordering of intervention modules or segments − Repeating elements or modules − Shortening/condensing	− Condensed treatment (each of the three main components were delivered in a single-session module) − Removed trauma narrative − Modules could be attended out of order or repeated	− Population changed to adolescents
Najavits et al., 2006 [58]	SS	USA	16.1-year-old (on average) girls with substance use disorder who experienced trauma	Improve fit	Not listed	− Adding elements − Lengthening/extending − Population − Tailoring/tweaking/refining	− Added two sessions for topics outside manual − Talked in displacement or discussed specific trauma details − Provided information verbally, if needed − Update given to parents, if agreed upon	− Population changed to adolescents
Schaeffer et al., 2013 [60]	MST-CAN and MST	USA	6–17-year-old children who experienced child maltreatment and are involved in child welfare	Improve fit	Not listed	− Integrating the intervention into another treatment approach − Population	− Integrated interventions into another treatment approach	− Population changed to children who experienced abuse/neglect
Swenson et al., 2010 [59]	MST	USA	13.9-year-old (on average) children who experienced physical abuse	Improve fit	Researcher	− Adding elements − Lengthening/extending − Population	− Lengthened treatment − Added pharmacotherapy (if needed) − Elements added to strengthen relationship between CPS and family	− Population changed to children who experienced physical abuse

### Trauma-Focused Cognitive Behavioral Therapy (TF-CBT)

Fifteen adaptations of TF-CBT were located [20–33, 61]. See Table 3. Commonly, who participated in the decision to adapt TF-CBT was not described [20, 22, 25, 31–33, 61]. Across all TF-CBT adaptations, the most common content adaptations were adding elements [20, 21, 23, 24, 26–29, 31–33, 61] and tailoring/tweaking/refining intervention components [21, 23, 25–27, 29, 30, 33], and the most common contextual adaptations were changes to format [21, 23, 25, 28, 29, 31–33] and personnel [26–29, 31, 32].

The most common reason for adaptation was to address cultural factors, with interventions being tested in Jordan [21], the Democratic Republic of Congo [25, 29], Zambia [26, 27], and with Hispanic children in the USA [30]. Given the aim of addressing cultural factors, many of these interventions engaged in tailoring/tweaking/refining to adapt language, examples, analogies, songs, or stories to fit with the culture in which they occurred [21, 25–27, 29, 30]. Several of these interventions made contextual adaptations by switching the format of treatment to group delivery [21, 25, 29]. Additionally, several interventions made changes to personnel, by adapting TF-CBT to be delivered by lay counselors [26, 27, 29], which was an element also tied to the goal of increasing the feasibility of these interventions in different cultural contexts where mental health clinicians may be less available.

TF-CBT was also adapted primarily to improve feasibility in other adaptations. In two instances, TF-CBT’s format was adapted to a stepped format, in which the first step was caregiver-led, with the second clinician-led step being only for those who did not respond to the first step of treatment [31, 32]. Another study shortened/condensed TF-CBT by reducing the number of sessions and removed/skipped elements by removing the trauma narrative to determine if these changes affected the efficacy of TF-CBT [22]. In a final adaptation to improve feasibility, the format (channel of treatment delivery) of TF-CBT was changed to be delivered through telehealth, with materials adapted to fit an electronic setting [33].

Finally, TF-CBT was adapted to improve fit [20, 23, 24, 28, 61]. In four instances, adaptations were related to traumatic loss [20, 24, 28, 61], with researchers in two studies [20, 28] making additional changes to a previous adaptation of TF-CBT to address traumatic loss [61]. Adaptations included adding elements to address traumatic loss [20, 24, 28, 61], condensing the number of grief module sessions [20], and removing parent sessions [24]. A further intervention aimed to improve fit with adolescents in the juvenile justice system by making adaptations to address the setting, such as tailoring/tweaking/refining the imaginal desensitization exercises. The typical format of parent interactions was also changed from in person to phone or videoconferencing, as these could not happen outside the correctional facility [23].

### Cognitive Behavioral Intervention for Trauma in Schools (CBITS)

Nine adaptations of CBITS were located [34–42]. See Table 4. In the majority of studies, adaptations were made in partnership with teams, including community members [34, 35, 37, 38, 40–42]. Across all CBITS adaptations, the most common content adaptations were tailoring/tweaking/refining [34–38, 40–42], adding elements [34–37, 40–42], and lengthening/extending [34, 35, 37, 38, 41, 42]. Contextual adaptations occurred equally across format [37, 39], setting [34, 35], personnel [34, 39], and population [34, 35].

A primary aim of five interventions was to address cultural factors, focusing on immigrant populations [35, 36, 38] and on American Indian populations [37, 40]. Adaptations varied, but most commonly content adaptations included tailoring/tweaking/refining to ensure material was culturally appropriate [35–38, 40], removing/skipping program components (e.g., introductory activities; [36, 40]), and adding/lengthening elements, such as family-related components [35, 36, 38]. An example of a contextual adaptation was changing the setting of the intervention from a school to the community [35].

The primary adaptation made in two studies was the addition of a family component that was designed not only to educate family members on CBITS, trauma, and helping their children through treatment, but was also designed to improve parent functioning [41, 42]. In contrast to CBITS, this family component was required and involved family members attending sessions where they were offered information on topics such as coping strategies and parenting [41, 42]. In both instances, tailoring/tweaking/refining took place to address cultural factors, with adaptations including the addition of culturally relevant examples; the intervention being lengthened or shortened depending on the needs of those involved; and materials from other approaches being integrated into treatment [41, 42].

One study adapted CBITS to create a new intervention – Support for Students Exposed to Trauma (SSET [39]). SSET is designed to improve the feasibility of CBITS by having the intervention be delivered by those without clinical training. Content adaptations involved the removing of sessions, with contextual adaptations outside who delivered the intervention involving changing from a session format to a lesson plan format and changing imaginal exposure to be in a curricular format [39]. SSET itself has now been classified as having promising research evidence by the California Evidence Based Clearinghouse [65].

One intervention was adapted to improve fit with females involved in child welfare. Content adaptations included removing the teacher session, adding sessions, lengthening sessions, and tailoring/tweaking/refining intervention components. Contextual adaptations included changing the setting of the intervention to be delivered in a mental health agency rather than a school, changing the target population, and changing the personnel to two group facilitators [34].

### Prolonged Exposure Therapy (PE)

Eight adaptations of PE were located [43–50]. See Table 5. The goal of all eight of these adaptations was to improve the fit of PE with an adolescent population [43–50]. Who made the decision to adapt the intervention was not listed in articles [43–50], though a manual [63] cited by study authors described adaptations being made by the research team, therapists, and community members in seven instances [43–49]. The same adaptations were made to tailor PE to adolescents in all but one [50] of the eight adaptations.

The seven studies following the same adaptations cited several sources for information on the adaptations made [47, 63, 66], but were often not explicit in the article itself about the adaptations made. Adaptations summarized here have been extracted from one of the manuals cited by study authors [63]. Adaptations included the addition of elements, including developmentally appropriate activities and the involvement of caregivers; tailoring/tweaking/refining intervention components, including procedures based on the emotional needs of the adolescent and simplifying language; and shortening session time when needed [43–49]. In the remaining article not following this manual, adaptations to PE were similar and included the addition of developmentally appropriate activities, which could be tailored based on the age of the child, and the involvement of caregivers in treatment [50].

Given the research supporting this new model, Prolonged Exposure Therapy for Adolescents (PE-A), is now considered to be well supported by the research evidence, the highest rating given by the CEBC [67].

### Narrative Exposure Therapy (NET)

Five adaptations of NET were located [51–55]. See Table 6. In all instances, contextual adaptations included changing the population of NET to a younger population, with the goal being to improve fit with that population, though no article described who participated in the decision to adapt the intervention [51–55]. Though all articles reported adapting NET to KIDNET, a new intervention for children developed from NET, the reported adaptations listed in articles varied. For example, though all articles mentioned adding illustrative, creative elements, such as the introduction of a string for the creation of a lifeline, stones, flowers, and drawings [51–55], just three described adding the use of body positioning to aid in the trauma reenactment [51, 54, 55]. KIDNET is now considered to have promising research evidence supporting its efficacy [68].

### Other EBIs

Two adaptations of Multisystemic Therapy (MST) were located [59, 60], with one being an adaptation of standard MST [59] and one being an adaptation of MST-CAN and MST [60]. See Table 7. Who participated in decisions to adapt was not listed in one study [60], while in the other researchers made the decision [59]. Researchers in both studies aimed to improve the fit of MST for children involved in the child welfare system [59, 60]. Content adaptations made to MST included lengthening treatment, adding elements to strengthen agency-family relationships, and adding pharmacotherapy when needed [59]. Content adaptations made to MST-CAN and MST involved integrating these interventions into another model (Reinforcement-Based Treatment) to create a new intervention [60].

One adaptation of SSET (an intervention derived from CBITS) was located. See Table 7. Both the research team and community members were involved in the adaptation. The main contextual adaptation was that those with clinical training led the intervention, though teachers served as co-facilitators. Content adaptations included the tailoring/tweaking/refining of intervention components, including translating materials, as well as the addition of regular parent meetings [56].

One adaptation of Skills Training in Affective and Interpersonal Regulation (STAIR), an intervention initially developed for adults, was located. See Table 7. STAIR was adapted by researchers and clinicians to improve fit with an adolescent, inpatient population. Specific adaptations included removing the trauma narrative, condensing treatment, giving adolescents the ability to repeat sessions, and re-ordering intervention components by giving adolescents the option to attend sessions out of order [57].

One adaptation of Seeking Safety (SS) was located (Table 7). Who participated in the adaptation was not explicated in the article, but the intervention, which was initially developed for adults, was adapted to improve fit with adolescent girls with substance use disorders. Adaptations included the addition of two sessions and updates to parents (if agreed upon), and tailoring/tweaking/refining material to talk in the displacement, discuss trauma details, and verbally deliver the material when needed [58].

## Discussion

### Overview of results

This review was the first comprehensive attempt to synthesize published literature on adaptations of EBIs for children who have experienced trauma. In total, 42 studies examining the adaptations of nine different EBIs were located, with TF-CBT and CBITS being the most commonly adapted EBIs. EBIs were most frequently adapted to improve fit with a new population and to address cultural factors. Most commonly, researchers in combination with others made decisions about adapting interventions, though who was involved in these decisions was often not described in articles. Common content adaptations included the addition of intervention elements and the tailoring/tweaking/refining of intervention materials. Common contextual adaptations included changes to the intended population, changes to the channel of treatment delivery, and changes to who administered the intervention.

### Implications and directions for future research

The most common reason for adaptation of EBIs was to improve fit with recipients. Specific need to improve fit often stemmed from needing to tailor an intervention to a new population (e.g., a younger population) or setting. This finding is in keeping with other systematic reviews on this topic where researchers found the need to adapt to a new population or setting to be the second and third most common reason for adaptation [7]. Though efforts have been made to adapt a number of EBIs to better fit with recipients, there have been a number of additional proposed adaptations to better fit the needs of LGBTQ youth [69], African American youth [70], and youth with autism spectrum disorder [71, 72] that should be evaluated.

The second most common reason for adapting an EBI was to address cultural factors. The need to adapt EBIs to new cultural contexts or to cultural groups is common and was the most common reason found for adapting public health interventions in a systematic review conducted by Escoffery, Lebow-Skelley [7]. Though interventions are increasingly being developed for, and adapted and evaluated in, multiple cultural contexts, many EBIs were developed in the USA and centered on majority (and often White) populations. There is a need to evaluate the efficacy of EBIs for diverse populations and contexts, and when necessary, develop adaptations for additional cultural contexts and populations to fully meet the needs of children who are experiencing trauma globally. For example, adaptations to EBIs to meet the cultural needs of children in China [73] have already been proposed. However, it is also critical to make decisions about adaptations deliberately based on the available research; for example, there is evidence that many EBIs are equally effective for children from different racial/ethnic backgrounds [74].

The reason for the adaptations guided the type of adaptation made. For example, when the reason for an intervention was a cultural adaptation, content adaptations were made that included adding culturally relevant material. In some instances, given resource constraints in some settings, contextual adaptations were made, including changing who administered the intervention (e.g., therapist to teacher). This practice of task shifting is a common occurrence in the delivery of mental health services [75], and is particularly important as the need for mental health services outpaces the capacity of mental health professionals in many countries and communities.

Methods for making decisions on adaptations were often not described, including whether they were made solely based on the authors’ expertise, existing research, needs assessments, consultation with community members, and/or pilot studies, which may be due to space or word limitations for journals. This represents a missed opportunity to share information about how successful adaptations are developed. Future researchers attempting to adapt interventions will benefit from partnering with those delivering and receiving the interventions when developing or evaluating adaptations to make to ensure that these adaptations fully meet the needs of those they are intended for. One avenue for more collaborative decision-making is a community-based participatory research (CBPR) approach [76] or user-centered design [77].

Though adaptations are frequently made to interventions, we must avoid the temptation to adapt every EBI for every unique setting or population without understanding how the original model works and for whom adaptations are necessary. Unnecessary adaptation may lead to an overwhelming number of variations with limited ability to understand their relative effectiveness or loss of the original EBI’s core components. This balance between intervention fidelity and adaptations is an ongoing challenge, but perhaps could be easier to navigate if the core components, opportunities for adaptation, and “red lines” for model fidelity (e.g., which adaptations are considered fidelity consistent or inconsistent) were more clearly articulated during EBI development. Often, EBI developers promote the flexibility of their model but also require strict fidelity, making it difficult to know how or when a model can be adapted. More explicit guidance and examples of successful fidelity-consistent adaptations can enhance efforts to scale and spread treatments effectively. Further, with limited resources and a growing number of trauma-focused EBIs, we must prioritize adaptations for populations where data indicate they are not being adequately served by existing EBIs. Research suggests that most EBIs are effective with diverse populations [78]. Specifically, existing EBIs should generally be considered a first line approach even with populations or children that may differ from the EBI research sample(s), even if with minor tailoring that maintains fidelity to the EBI. Use of outcome data on EBIs in practice with diverse populations should be used to inform the decision to develop adaptations.

Though this review published existing academic and grey literature on adaptations, the research on adaptations pales in comparison to how frequently adaptations are likely made in real-world settings (but never evaluated or published). In light of the emphasis on fidelity during training and implementation, as well as possible implicit or explicit power differentials between practitioners and those who conduct and champion the research or implementation, providers may be reluctant to share these adaptations with EBI developers for fear of running afoul of treatment fidelity or certification requirements. This is another reason that further research with practitioners in real-world settings (e.g., using CBPR) is needed to determine what adaptations are being made in practice to EBIs and how these adaptations may affect intervention outcomes and access to services.

### Limitations

It is possible that studies relevant to this review were not located. Specifically, though we did not limit our searches to the English language, we only included studies published in the English language. Further, we excluded studies of EBIs that included people over the age of 18, even in instances where some of the population were 18 or under (e.g., [79–85]). Finally, it is possible that studies with non-significant results were not published due to publication bias. These studies may have provided additional insight into adaptations of EBIs for children.

The synthesis of adaptations made is also limited by the source articles. Due to space constraints, it is possible that the study authors did not fully report on all aspects of the adaptation process, including who was involved in the adaptations and all of the adaptations that were made. If aspects of adaptation were omitted from articles, these would not have been extracted by our research team, and thus would not be synthesized here. Some of the information missing from articles may have been available in published implementation manuals, however, due to funding constraints, the research team was unable to extract data from these sources, with the exception of the PE-A manual.

## Conclusion

This systematic review found that EBIs have been frequently adapted and evaluated for use with children globally. Most published studies of EBI adaptions have been developed to improve fit and address cultural factors. However, little research is available about adaptations made by clinicians in day-to-day practice. Efforts should be made to evaluate the various types of adaptations and especially whether adaptations improve access to services or child outcomes in order to ensure that all children exposed to trauma can access effective treatment.

## Supplementary Information

**Additional file 1.** PRISMA 2020 Checklist.

**Additional file 2.** Search Strategy.

**Additional file 3.** Study Quality.

## Data Availability

All data generated or analyzed during this study are included in this published article and its supplementary information files.

## Abbreviations

CBITS: Cognitive Behavioral Intervention for Trauma in Schools
CBT-CTG: Cognitive Behavioral Therapy for Childhood Traumatic Grief
CPS: Child Protective Services
DRC: Democratic Republic of Congo
EBI: Evidence-based intervention
MPE: Modified Prolonged Exposure
MST: Multisystemic Therapy
MST-CAN: Multisystemic Therapy for Child Abuse and Neglect
NET: Narrative Exposure Therapy
PE: Prolonged Exposure
PE-A: Prolonged Exposure Therapy for Adolescents
PTEs: Potentially traumatic events
SS: Seeking Safety
SSET: Support for Students Exposed to Trauma
STAIR: Skills Training in Affective and Interpersonal Regulation
TF-CBT: Trauma-Focused Cognitive Behavioral Therapy
USA: United States of America

## References

[1] Copeland WE, Keeler G, Angold A, Costello EJ (2007). Traumatic events and posttraumatic stress in childhood. Arch Gen Psychiatry.

[2] McLaughlin KA (2016). Future directions in childhood adversity and youth psychopathology. J Clin Child Adolesc Psychol.

[3] Centers for Disease Control and Prevention (2021). Adverse Childhood Experiences (ACEs).

[4] Finkelhor D, Turner HA, Shattuck A, Hamby SL (2015). Prevalence of childhood exposure to violence, crime, and abuse: Results from the national survey of children’s exposure to violence. JAMA Pediatr.

[5] Hughes K, Bellis MA, Hardcastle KA, Sethi D, Butchart A, Mikton C (2017). The effect of multiple adverse childhood experiences on health: A systematic review and meta-analysis. Lancet Public Health.

[6] Leenarts LE, Diehle J, Doreleijers TA, Jansma EP, Lindauer RJ (2013). Evidence-based treatments for children with trauma-related psychopathology as a result of childhood maltreatment: A systematic review. Eur Child Adolesc Psychiatry.

[7] Escoffery C, Lebow-Skelley E, Haardoerfer R, Boing E, Udelson H, Wood R (2018). A systematic review of adaptations of evidence-based public health interventions globally. Implement Sci.

[8] Dorsey S, McLaughlin KA, Kerns SE, Harrison JP, Lambert HK, Briggs EC (2017). Evidence base update for psychosocial treatments for children and adolescents exposed to traumatic events. J Clin Child Adolesc Psychol.

[9] Wethington HR, Hahn RA, Fuqua-Whitley DS, Sipe TA, Crosby AE, Johnson RL (2008). The effectiveness of interventions to reduce psychological harm from traumatic events among children and adolescents: A systematic review. Am J Prev Med.

[10] Hambrick EP, Oppenheim-Weller S, N'zi AM, Taussig HN. (2016). Mental health interventions for children in foster care: A systematic review. Children and Youth Services Review.

[11] Howarth E, Moore TH, Welton NJ, Lewis N, Stanley N, MacMillan H (2016). IMPRoving Outcomes for children exposed to domestic ViolencE (IMPROVE): An evidence synthesis. Public Health Res.

[12] Lange B, Nelson A, Lang J, Stirman S (2020). A systematic review of adaptations of evidence-based interventions to address traumatic events among children and adolescents PROSPERO.

[13] Page MJ, McKenzie JE, Bossuyt PM, Boutron I, Hoffmann TC, Mulrow CD (2021). The PRISMA 2020 statement: An updated guideline for reporting systematic reviews. Int J Surg.

[14] The National Child Traumatic Stress Network (2019). Interventions.

[15] Ouzzani M, Hammady H, Fedorowicz Z, Elmagarmid A (2016). Rayyan—a web and mobile app for systematic reviews. Syst Rev.

[16] California Evidence-Based Clearinghouse for Child Welfare (2022). Programs.

[17] Blueprints for Healthy Youth Development (2022). Program search.

[18] Stirman SW, Baumann AA, Miller CJ (2019). The FRAME: An expanded framework for reporting adaptations and modifications to evidence-based interventions. Implement Sci.

[19] Kennedy CE, Fonner VA, Armstrong KA, Denison JA, Yeh PT, O’Reilly KR (2019). The Evidence Project risk of bias tool: Assessing study rigor for both randomized and non-randomized intervention studies. Syst Rev.

[20] Cohen JA, Mannarino AP, Staron VR (2006). A pilot study of modified cognitive-behavioral therapy for childhood traumatic grief (CBT-CTG). J Am Acad Child Adolesc Psychiatry.

[21] Damra JKM, Nassar YH, Ghabri TMF (2014). Trauma-focused cognitive behavioral therapy: Cultural adaptations for application in Jordanian culture. Couns Psychol Quart.

[22] Deblinger E, Mannarino AP, Cohen JA, Runyon MK, Steer RA (2011). Trauma-focused cognitive behavioral therapy for children: Impact of the trauma narrative and treatment length. Depress Anxiety.

[23] Heier JE (2018). Capturing fidelity to understand implementation of Trauma-focused Cognitive Behavioral Therapy in juvenile justice correctional facilities.

[24] Madigan S, Vaillancourt K, McKibbon A, Benoit D (2015). Trauma and traumatic loss in pregnant adolescents: The impact of Trauma-Focused Cognitive Behavior Therapy on maternal unresolved states of mind and Posttraumatic Stress Disorder. Attach Hum Dev.

[25] McMullen J, O'callaghan P, Shannon C, Black A, Eakin J (2013). Group trauma-focused cognitive-behavioural therapy with former child soldiers and other war-affected boys in the DR Congo: A randomised controlled trial. J Child Psychol Psychiatry.

[26] Murray LK, Familiar I, Skavenski S, Jere E, Cohen J, Imasiku M (2013). An evaluation of trauma focused cognitive behavioral therapy for children in Zambia. Child Abuse Negl.

[27] Murray LK, Skavenski S, Kane JC, Mayeya J, Dorsey S, Cohen JA (2015). Effectiveness of trauma-focused cognitive behavioral therapy among trauma-affected children in Lusaka, Zambia: A randomized clinical trial. JAMA Pediatr.

[28] O'Donnell K, Dorsey S, Gong W, Ostermann J, Whetten R, Cohen JA (2014). Treating maladaptive grief and posttraumatic stress symptoms in orphaned children in Tanzania: Group-based trauma-focused cognitive–behavioral therapy. J Trauma Stress.

[29] O’Callaghan P, McMullen J, Shannon C, Rafferty H, Black A (2013). A randomized controlled trial of trauma-focused cognitive behavioral therapy for sexually exploited, war-affected Congolese girls. J Am Acad Child Adolesc Psychiatry.

[30] Rivera S (2008). Culturally-modified trauma-focused treatment for Hispanic children: Preliminary findings: St. Mary's University.

[31] Salloum A, Robst J, Scheeringa MS, Cohen JA, Wang W, Murphy TK (2014). Step one within stepped care trauma-focused cognitive behavioral therapy for young children: A pilot study. Child Psychiatry Hum Dev.

[32] Salloum A, Small BJ, Robst J, Scheeringa MS, Cohen JA, Storch EA (2017). Stepped and standard care for childhood trauma: A pilot randomized clinical trial. Res Soc Work Pract.

[33] Stewart RW, Orengo-Aguayo RE, Cohen JA, Mannarino AP, de Arellano MA (2017). A pilot study of trauma-focused cognitive–behavioral therapy delivered via telehealth technology. Child Maltreat.

[34] Auslander W, McGinnis H, Tlapek S, Smith P, Foster A, Edmond T (2017). Adaptation and implementation of a trauma-focused cognitive behavioral intervention for girls in child welfare. Am J Orthopsychiatry.

[35] Elswick S, Washington G, Mangrum-Apple H, Peterson C, Barnes E, Pirkey P, et al. Trauma Healing Club: Utilizing culturally responsive processes in the implementation of an after-school group intervention to address trauma among African refugees. J Child Adolesc Trauma. 2021:1–12. 10.1007/s40653-021-00387-5 PMC8302980 34336082

[36] Feldman ES (2007). Implementation of the cognitive behavioral intervention for trauma in schools (CBITS) with Spanish-speaking, immigrant middle-school students: Is effective, culturally competant treatment possible within a public school setting?.

[37] Goodkind JR, LaNoue MD, Milford J (2010). Adaptation and implementation of cognitive behavioral intervention for trauma in schools with American Indian youth. J Clin Child Adolesc Psychol.

[38] Kataoka SH, Stein BD, Jaycox LH, Wong M, Escudero P, Tu W (2003). A school-based mental health program for traumatized Latino immigrant children. J Am Acad Child Adolesc Psychiatry.

[39] Jaycox LH, Langley AK, Stein BD, Wong M, Sharma P, Scott M (2009). Support for students exposed to trauma: A pilot study. School Mental Health.

[40] Morsette A, Swaney G, Stolle D, Schuldberg D, van den Pol R, Young M (2009). Cognitive behavioral intervention for trauma in schools (CBITS): School-based treatment on a rural American Indian reservation. J Behav Ther Exp Psychiatry.

[41] Santiago CD, Lennon JM, Fuller AK, Brewer SK, Kataoka SH (2014). Examining the impact of a family treatment component for CBITS: When and for whom is it helpful?. J Fam Psychol.

[42] Santiago CD, Kataoka SH, Hu-Cordova M, Alvarado-Goldberg K, Maher LM, Escudero P (2015). Preliminary evaluation of a family treatment component to augment a school-based intervention serving low-income families. J Emot Behav Disord.

[43] Aderka IM, Foa EB, Applebaum E, Shafran N, Gilboa-Schechtman E (2011). Direction of influence between posttraumatic and depressive symptoms during prolonged exposure therapy among children and adolescents. J Consult Clin Psychol.

[44] Aderka IM, Appelbaum-Namdar E, Shafran N, Gilboa-Schechtman E (2011). Sudden gains in prolonged exposure for children and adolescents with posttraumatic stress disorder. J Consult Clin Psychol.

[45] Brown LA, Belli G, Suzuki N, Capaldi S, Foa EB. Reduction in suicidal ideation from prolonged exposure therapy for adolescents. J Clin Child Adolesc Psychol. 2019:1–9. 10.1080/15374416.2019.1614003 PMC6885098 31150295

[46] Foa E, McLean C, Capaldi S, Rosenfield D (2013). Prolonged exposure vs supportive counseling for sexual abuse–related PTSD in adolescent girls: A randomized clinical trial. JAMA..

[47] Gilboa-Schechtman E, Foa EB, Shafran N, Aderka IM, Powers MB, Rachamim L (2010). Prolonged exposure versus dynamic therapy for adolescent PTSD: A pilot randomized controlled trial. J Am Acad Child Adolesc Psychiatry.

[48] McLean CP, Yeh R, Rosenfield D, Foa EB (2015). Changes in negative cognitions mediate PTSD symptom reductions during client-centered therapy and prolonged exposure for adolescents. Behav Res Ther.

[49] McLean CP, Su Y-J, Carpenter JK, Foa EB (2017). Changes in PTSD and depression during prolonged exposure and client-centered therapy for PTSD in adolescents. J Clin Child Adolesc Psychol.

[50] Adler Nevo G, Manassis K (2011). An adaptation of prolonged exposure therapy for pediatric single incident trauma: A case series. J Can Acad Child Adolesc Psychiatry.

[51] Catani C, Kohiladevy M, Ruf M, Schauer E, Elbert T, Neuner F (2009). Treating children traumatized by war and Tsunami: A comparison between exposure therapy and meditation-relaxation in North-East Sri Lanka. BMC Psychiatry.

[52] Onyut LP, Neuner F, Schauer E, Ertl V, Odenwald M, Schauer M (2005). Narrative Exposure Therapy as a treatment for child war survivors with posttraumatic stress disorder: Two case reports and a pilot study in an African refugee settlement. BMC Psychiatry.

[53] Peltonen K, Kangaslampi S (2019). Treating children and adolescents with multiple traumas: A randomized clinical trial of narrative exposure therapy. Eur J Psychotraumatol.

[54] Ruf M, Schauer M, Neuner F, Catani C, Schauer E, Elbert T (2010). Narrative exposure therapy for 7-to 16-year-olds: A randomized controlled trial with traumatized refugee children. J Trauma Stress.

[55] Schauer E (2008). Trauma treatment for children in war: Build-up of an evidence-based large-scale mental health intervention in north-eastern Sri Lanka.

[56] Amin R, Nadeem E, Iqbal K, Asadullah MA, Hussain B (2020). Support for Students Exposed to Trauma (SSET) Program: An approach for building resilience and social support among flood-impacted children. School Mental Health.

[57] Gudiño OG, Weis JR, Havens JF, Biggs EA, Diamond UN, Marr M (2014). Group trauma-informed treatment for adolescent psychiatric inpatients: A preliminary uncontrolled trial. J Trauma Stress.

[58] Najavits LM, Gallop RJ, Weiss RD (2006). Seeking safety therapy for adolescent girls with PTSD and substance use disorder: A randomized controlled trial. J Behav Health Serv Res.

[59] Swenson CC, Schaeffer CM, Henggeler SW, Faldowski R, Mayhew AM (2010). Multisystemic therapy for child abuse and neglect: A randomized effectiveness trial. J Fam Psychol.

[60] Schaeffer CM, Swenson CC, Tuerk EH, Henggeler SW (2013). Comprehensive treatment for co-occurring child maltreatment and parental substance abuse: Outcomes from a 24-month pilot study of the MST-Building Stronger Families program. Child Abuse Negl.

[61] Cohen JA, Mannarino AP, Knudsen K (2004). Treating childhood traumatic grief: A pilot study. J Am Acad Child Adolesc Psychiatry.

[62] Schauer M, Neuner F, Elbert T (2017). Narrative exposure therapy for children and adolescents (KIDNET). Evidence-based treatments for trauma related disorders in children and adolescents.

[63] Foa E, Chrestman KR, Gilboa-Schechtman E (2008). Prolonged exposure therapy for adolescents with PTSD emotional processing of traumatic experiences, therapist guide.

[64] Neuner F, Catani C, Ruf M, Schauer E, Schauer M, Elbert T (2008). Narrative Exposure Therapy for the treatment of child and adolescent war victims: From neurobiology to field intervention. Child Adolesc Psychiatr Clin N Am.

[65] California Evidence-Based Clearinghouse for Child Welfare (2020). Support for Students Exposed to Trauma (SSET).

[66] Foa E, Chrestman K, Gilboa-Schechtman E (2008). Prolonged exposure manual for children and adolescents suffering from PTSD.

[67] California Evidence-Based Clearinghouse for Child Welfare (2018). Prolonged Exposure Therapy for Adolescents (PE-A).

[68] California Evidence-Based Clearinghouse for Child Welfare (2018). KIDNET.

[69] Cohen JA, Marnnarino A, Wilson K, Zinny A (2018). Implementing Trauma-Focused Cognitive Behavioral Therapy for LGBTQ youth and their caregivers.

[70] Metzger IW, Anderson RE, Are F, Ritchwood T (2021). Healing interpersonal and racial trauma: Integrating racial socialization into trauma-focused cognitive behavioral therapy for African American youth. Child Maltreat.

[71] Peterson JL, Earl RK, Fox EA, Ma R, Haidar G, Pepper M (2019). Trauma and autism spectrum disorder: Review, proposed treatment adaptations and future directions. J Child Adolesc Trauma.

[72] Romney JS, Garcia M. TF-CBT informed teletherapy for children with autism and their families. J Child Adolesc Trauma. 2021;14:415–24. 10.1007/s40653-021-00354-0 PMC8056103 33897936

[73] Li J, Li J, Yuan L, Zhou Y, Qu Z (2020). Cultural adaptation and feasibility of trauma-focused cognitive behavioural therapy in China.

[74] Huey SJ, Polo AJ (2008). Evidence-based psychosocial treatments for ethnic minority youth. J Clin Child Adolesc Psychol.

[75] Javadi D, Feldhaus I, Mancuso A, Ghaffar A. Applying systems thinking to task shifting for mental health using lay providers: a review of the evidence. Glob Mental Health. 2017;4. 10.1017/gmh.2017.15 PMC5719475 29230310

[76] Baum F, MacDougall C, Smith D (2006). Participatory action research. J Epidemiol Community Health.

[77] Lyon AR, Koerner K (2016). User-centered design for psychosocial intervention development and implementation. Clin Psychol Sci Pract.

[78] Lang JM, Lee P, Connell CM, Marshall T, Vanderploeg JJ (2021). Outcomes, evidence-based treatments, and disparities in a statewide outpatient children’s behavioral health system. Children and Youth Services Review.

[79] Ertl V, Pfeiffer A, Schauer E, Elbert T, Neuner F (2011). Community-implemented trauma therapy for former child soldiers in Northern Uganda: A randomized controlled trial. JAMA..

[80] Hermenau K, Hecker T, Schaal S, Maedl A, Elbert T (2013). Addressing post-traumatic stress and aggression by means of narrative exposure: A randomized controlled trial with ex-combatants in the Eastern DRC. J Aggress Maltreat Trauma.

[81] Schaal S, Elbert T, Neuner F (2009). Narrative exposure therapy versus interpersonal psychotherapy. Psychother Psychosom.

[82] Wang DC, Aten JD, Boan D, Jean-Charles W, Griff KP, Valcin VC (2016). Culturally adapted spiritually oriented trauma-focused cognitive–behavioral therapy for child survivors of restavek. Spirituality Clin Pract.

[83] Robjant K, Koebach A, Schmitt S, Chibashimba A, Carleial S, Elbert T (2019). The treatment of posttraumatic stress symptoms and aggression in female former child soldiers using adapted narrative exposure therapy–a RCT in Eastern Democratic Republic of Congo. Behav Res Ther.

[84] Peters W, Rice S, Cohen J, Murray L, Schley C, Alvarez-Jimenez M (2021). Trauma-focused cognitive–behavioral therapy (TF-CBT) for interpersonal trauma in transitional-aged youth. Psychol Trauma Theory Res Pract Policy.

[85] Vogel A, Rosner R (2020). Lost in transition? Evidence-based treatments for adolescents and young adults with posttraumatic stress disorder and results of an uncontrolled feasibility trial evaluating cognitive processing therapy. Clin Child Fam Psychol Rev.

