# Supplementary material for: Adaptations of evidence-based trauma-focused interventions for children and adolescents: a systematic review
Source: Implement Sci Commun. 2022 Oct 8;3:108. doi: 10.1186/s43058-022-00348-5 (PMC9548160; doi:10.1186/s43058-022-00348-5)
Supplement: Supplementary file 2 — Additional file 2. Search Strategy. [file 43058_2022_348_MOESM2_ESM.docx]

**Appendix 1: Search Strategy**

**Search Strategy – Applied Social Sciences Index & Abstracts (ASSIA)**

**Search date:** October 14, 2019

**Search strategy, as it appears in the database:**

(AB(intervention*) OR AB(therap*) OR AB(psychotherap*) OR AB(treatment*) OR AB(trial*) OR SU(interventions) OR SU(cognitive behaviour therapy) OR SU(cognitive behavioural psychotherapy) OR SU(psychotherapy) OR SU(treatment)) AND (AB(trauma*) OR SU(traumatic incidents) OR SU(traumatic life events) OR SU(physical trauma) OR SU(psychological trauma)) AND (AB(toddler*) OR AB(child*) OR AB(adolescent*) OR AB(youth*) OR AB(boy*) OR AB(girl*) OR AB(student*) OR SU(children) OR SU(adolescent)) AND (AB(adapt*) OR AB(adjust*) OR AB(modif*) OR SU(adaptation) OR SU(modification))

**Notes:** There were 982 hits, but only 975 downloaded (duplicate articles)

**Embase**

**Search date:** October 14, 2019

**Search strategy, as it appears in the database:**

|  | **Searches** | **Results** | **Type** |
| --- | --- | --- | --- |
| 1 | (Intervention* or Therap* or Psychotherap* or Treatment* or Trial*).ab. or Intervention study.sh. or Behavior therapy.sh. or Cognitive therapy.sh. | 8156589 | Advanced |
| 2 | Trauma*.ab. | 390266 | Advanced |
| 3 | (Toddler* or Child* or Adolescent* or Youth* or Boy* or Girl* or Student*).ab. or Toddler.sh. or Preschool child.sh. or Child.sh. or Adolescent.sh. | 3365940 | Advanced |
| 4 | (Adapt* or Adjust* or Modif*).ab. or Adaptation.sh. | 2472074 | Advanced |
| 5 | 1 and 2 and 3 and 4 | 3840 | Advanced |

**Notes:** None

**ProQuest Dissertations & Theses Global: Global Full Text**

**Search date:** October 14, 2019

**Search strategy, as it appears in the database:**

(AB(intervention*) OR AB(therap*) OR AB(psychotherap*) OR AB(treatment*) OR AB(trial*)) AND AB(trauma*) AND (AB(toddler*) OR AB(child*) OR AB(adolescent*) OR AB(youth*) OR AB(boy*) OR AB(girl*) OR AB(student*) ) AND (AB(adapt*) OR

AB(adjust*) OR AB(modif*))

**Notes:** None

**PsycINFO**

**Search date:** October 14, 2019

**Search strategy, as it appears in the database:**

|  | **Searches** | **Results** | **Type** |
| --- | --- | --- | --- |
| 1 | (Intervention* or Therap* or Psychotherap* or Treatment* or Trial*).ab. or Intervention.sh. or Cognitive therapy.sh. or Psychotherapy.sh. or Treatment.sh. | 1128173 | Advanced |
| 2 | Trauma*.ab. or Trauma.sh. or Emotional Trauma.sh. | 101324 | Advanced |
| 3 | (Toddler* or Child* or Adolescent* or Youth* or Boy* or Girl* or Student*).ab. | 1176884 | Advanced |
| 4 | (Adapt* or Adjust* or Differential effect* or Modif*).ab. or Adaptation.sh. | 408265 | Advanced |
| 5 | 1 and 2 and 3 and 4 | 2122 | Advanced |

**Notes:** None

**PubMed**

**Original search date:** October 14, 2019

**Supplementary search date:** August 31, 2021

**Search strategy, as it appears in the database:**

((((“Intervention*” [Text Word] OR “Therap*” [Text Word] OR “Psychotherap*” [Text Word] OR “Treatment*” [Text Word] OR “Trial*” [Text Word] OR “Cognitive behavioral therapy” [MeSH Terms] OR “Psychotherapy” [MeSH Terms])) AND (“Trauma*” [Text Word] OR “Psychological trauma” [MeSH Terms])) AND (“Toddler*” [Text Word] OR “Child*” [Text Word] OR “Adolescent*” [Text Word] OR “Youth*” [Text Word] OR “Boy*” [Text Word] OR “Girl*” [Text Word] OR “Student*” [Text Word] OR “Child” [MeSH Terms] OR “Adolescent” [MeSH Terms])) AND (“Adapt*” [Text Word] OR “Adjust*” [Text Word] OR “Modif*” [Text Word])

**Notes:** None

**Scopus**

**Search date:** October 14, 2019

**Search strategy, as it appears in the database:**

( TITLE ( intervention* OR therap* OR psychotherap* OR treatment* OR trial* ) AND TITLE-ABS-KEY ( trauma* ) AND TITLE-ABS-KEY ( toddler* OR child* OR adolescent* OR youth* OR boy* OR girl* OR student* ) AND TITLE-ABS-KEY ( adapt* OR adjust* OR modif* ) )

**Notes:** None

**Social Services Abstracts**

**Search date:** October 14, 2019

**Search strategy, as it appears in the database:**

(AB(intervention*) OR AB(therap*) OR AB(psychotherap*) OR AB(treatment*) OR AB(trial*) OR SU(intervention) OR SU(psychotherapy) OR SU(treatment)) AND (AB(trauma*) OR SU(trauma)) AND (AB(toddler*) OR AB(child*) OR AB(adolescent*) OR AB(youth*) OR AB(boy*) OR AB(girl*) OR AB(student*) OR SU(children) OR SU(adolescents)) AND (AB(adapt*) OR AB(adjust*) OR AB(modif*))

**Notes:** 1,025 hits, but 1,017 downloaded (duplicates)

**Sociological Abstracts**

**Search date:** October 14, 2019

**Search strategy, as it appears in the database:**

(AB(intervention*) OR AB(therap*) OR AB(psychotherap*) OR AB(treatment*) OR AB(trial*) OR SU(intervention) OR SU(psychotherapy) OR SU(treatment)) AND (AB(trauma*) OR SU(trauma)) AND (AB(toddler*) OR AB(child*) OR AB(adolescent*) OR AB(youth*) OR AB(boy*) OR AB(girl*) OR AB(student*) OR SU(children) OR SU(adolescents)) AND (AB(adapt*) OR AB(adjust*) OR AB(modif*))

**Notes:** 411 hits, but 407 downloaded (duplicates)

**Web of Science**

**Search date:** October 14, 2019

**Search strategy, as it appears in the database:**

TITLE: (Intervention* OR Therap* OR Psychotherap* OR Treatment* OR Trial*) AND TOPIC: (Trauma*) AND TOPIC: (Toddler* OR Child* OR Adolescent* OR Youth* OR Boy* OR Girl* OR Student*) AND TOPIC: (Adapt* OR Adjust* OR Modif*)

**Notes:**

“All Databases” was selected when running this search. Therefore, the following databases were included within this search:

- Web of Science^TM^ Core Collection (1945-present)
  - Science Citation Index Expanded (1945-present)
  - Social Sciences Citation Index (1956-present)
  - Arts & Humanities Citation Index (1975-present)
  - Conference Proceedings Citation Index- Science (1990-present)
  - Conference Proceedings Citation Index- Social Science & Humanities (1990-present)
  - Book Citation Index– Science (2005-present)
  - Book Citation Index– Social Sciences & Humanities (2005-present)
  - Emerging Sources Citation Index (2015-present)
  - Current Chemical Reactions (1986-present)
  - Index Chemicus (1993-present)
- BIOSIS Citation Index^SM^ (1969-present)
- Current Contents Connect® (1998-present)
- Data Citation Index^SM^ (1993-present)
- Derwent Innovations Index^SM^ (1993-present)
- Inspec® (1969-present)
- KCI-Korean Journal Database (1980-present)
- MEDLINE® (1950-present)
- Russian Science Citation Index (2005-present)
- SciELO Citation Index (1997-present)
- Zoological Record® (1993-present)
