# Supplementary material for: Adaptations of evidence-based trauma-focused interventions for children and adolescents: a systematic review
Source: Implement Sci Commun. 2022 Oct 8;3:108. doi: 10.1186/s43058-022-00348-5 (PMC9548160; doi:10.1186/s43058-022-00348-5)
Supplement: Supplementary file 3 — Additional file 3. Study Quality. [file 43058_2022_348_MOESM3_ESM.docx]

**Appendix 2: Study Quality Ratings**

| **Study Author (Year)** | **Cohort** | | | **Participant Representativeness** | | | **Equivalence of Comparison Groups** | |
| --- | --- | --- | --- | --- | --- | --- | --- | --- |
|  | **Cohort** | **Control or comparison group** | **Pre-post intervention data** | **Random assignment of participants to the intervention** | **Random selection of participants for assessment** | **Follow-up rate of 80% or more** | **Comparison groups equivalent on socio-demographics** | **Comparison groups equivalent at baseline on outcome measures** |
| **Trauma-Focused Cognitive Behavioral Therapy** | | | | | | | | |
| Cohen et al, 2004 | Yes | No | Yes | N/A | No | Yes | N/A | N/A |
| Cohen et al., 2006 | Yes | No | Yes | N/A | No | No | N/A | N/A |
| Damra et al., 2014 | Yes | Yes | Yes | Yes | No | Yes | NR, but all were male | Yes, on all but PTSD |
| Deblinger et al., 2011 | Yes | Yes | Yes | Yes | No | No | Yes | Yes |
| Heier, 2018 | Yes | Yes, but not used in analysis | Yes | No | No | No | N/A | N/A |
| Madigan et al., 2015 | Yes | Yes | Yes | Yes | No | No | Yes | Yes |
| McMullen et al., 2013 | Yes | Yes | Yes | Yes | No | No | NR, but all were male | Yes |
| Murray et al., 2013 | Yes | No | Yes | N/A | No | No | N/A | N/A |
| Murray et al., 2015 | Yes | Yes | Yes | Yes | No | Yes | Yes | Yes |
| O’Callaghan et al., 2013 | Yes | Yes | Yes | Yes | No | Yes | Yes | Yes |
| O'Donnell et al., 2014 | Yes | No | Yes | N/A | No | Yes | N/A | N/A |
| Rivera, 2008 | Yes | No | Yes | N/A | No | Yes | N/A | N/A |
| Salloum et al., 2014 | Yes | No | Yes | N/A | No | No | N/A | N/A |
| Salloum et al., 2017 | Yes | Yes | Yes | Yes | No | No | Yes, on all but parent age | NR |
| Stewart et al., 2017 | Yes | No | Yes | N/A | No | Yes | N/A | N/A |
| **Cognitive Behavioral Intervention for Trauma in Schools** | | | | | | | | |
| Auslander et al., 2017 | Yes | Yes | Yes | Yes | No | Yes | NR | NR |
| Elswick, 2021 | Yes | No | Yes | N/A | No | Yes | N/A | N/A |
| Feldman, 2007 | Yes | No | Yes | N/A | No | No | N/A | N/A |
| Goodkind et al., 2010 | Yes | No | Yes | N/A | No | Yes | N/A | N/A |
| Jaycox et al., 2009 | Yes | Yes | Yes | Yes | No | Yes | Yes, on all but % Hispanic | NR |
| Kataoka et al., 2003 | Yes | Yes | Yes | Yes | No | No | Yes, on all but parent education | Yes |
| Morsette et al., 2009 | Yes | No | Yes | N/A | No | No | N/A | N/A |
| Santiago et al., 2014 | Yes | Yes | Yes | No | No | No | Yes | NR |
| Santiago et al., 2015 | Yes | Yes | Yes | No | No | Yes | Yes | NR |
| **Prolonged Exposure Therapy** | | | | | | | | |
| Aderka, Appelbaum-Namdar, et al., 2011 | Yes | No | Yes | N/A | No | No | N/A | N/A |
| Aderka, Foa, et al., 2011 | Yes | No | Yes | N/A | No | NR | N/A | N/A |
| Adler Nevo & Manassis, 2011 | Yes | No | Yes | N/A | No | Yes | N/A | N/A |
| Brown, 2019 | Yes | Yes | Yes | Yes | No | Yes | Yes | Yes |
| Foa et al., 2013 | Yes | Yes | Yes | Yes | No | Yes | Yes | Yes |
| Gilboa-Schechtman et al., 2010 | Yes | Yes | Yes | Yes | No | No | Yes | Yes |
| McLean et al., 2015 | Yes | Yes | Yes | Yes | No | Yes | NR | NR |
| McLean et al., 2017 | Yes | Yes | Yes | Yes | No | Yes | NR | Yes |
| **Narrative Exposure Therapy** | | | | | | | | |
| Catani et al. 2009 | Yes | Yes | Yes | Yes | No | Yes | Yes | Yes |
| Onyut et al., 2005 | Yes | No | Yes | N/A | No | Yes | N/A | N/A |
| Peltonen & Kangaslampi, 2019 | Yes | Yes | Yes | Yes | No | No | Yes | Yes |
| Ruf et al., 2010 | Yes | Yes | Yes | Yes | No | Yes | Yes | Yes |
| Schauer, 2008 | Yes | Yes | Yes | Yes | No | Yes | Yes | Yes |
| **Other EBIs** | | | | | | | | |
| Amin, 2020 | Yes | Yes | Yes | Yes | No | Yes | Yes | Yes |
| Gudiño et al., 2014 | Yes | No | Yes | N/A | No | Yes | N/A | N/A |
| Najavits et al., 2006 | Yes | Yes | Yes | Yes | No | No | Yes | Yes |
| Schaeffer et al., 2013 | Yes | Yes | Yes | No | No | Yes | Yes | NR |
| Swenson et al., 2010 | Yes | Yes | Yes | Yes | No | Yes | Yes | Yes |

Abbreviations:

- N/A: Not applicable
- NR: Not reported
